# PReS-FINAL-2062: The need for conducting different rehabilitation complexes for patients with juvenile idiopathic arthritis (JIA)

**DOI:** 10.1186/1546-0096-11-S2-P74

**Published:** 2013-12-05

**Authors:** TA Shelepina, IP Nikishina

**Affiliations:** 1 Pediatric Rheumatology, Scientific Research Institute of Rheumatology, Moscow, Russian Federation

## Introduction

We have developed and implemented rehabilitation complexes. *A corrective complex *- for disabled patients with severe functional restrictions, causing social constraints. *A mobilization complex *- for patients with moderate functional restrictions that do not cause social constraints. *A health-improving complex *- for functionally intact patients with no functional restrictions.

## Objectives

to compare the need for such complexes in patients, who were in hospital in 2007, 2008 and 2012.

## Methods

Children's ward patients suffering from various forms of JIA, aged two to eighteen years, who were in the hospital in 2007, 2008 and 2012 and underwent rehabilitation treatment; over 50% of the patients were re-hospitalized within a specified time. We compared the need for conducting different rehabilitation treatment complexes in patients treated in 2007, 2008 and 2012.

## Results

See Table 1.

**Table 1:** 

Rehabilitation complexes	2007	2008	2012
Corrective	6 (3%)	8 (4%)	4 (2%)

Mobilization	140 (67%)	133 (65%)	106 (49%)

Health-improving	65 (30%)	64 (31%)	105 (49%)

Total number of patients	210	205	215

## Conclusion

A statistically significant increase was detected in the number of functionally intact patients among the patients undergoing rehabilitation treatment in 2012, compared with those undergoing treatment in 2007, and in 2008, thanks to the practical introduction of biologic agents. The total number of patients in need of rehabilitation treatment remains high.

## Disclosure of interest

None declared.

